# Synergistic mitigation of endotoxin-induced liver injury by low-frequency PMF and 27.12 MHz RF-EMF: a multi-biomarker experimental study

**DOI:** 10.1007/s00068-026-03119-2

**Published:** 2026-02-23

**Authors:** Bilal Turan, Halil Asci, Orhan Imeci, Muhammet Tepebasi, Arzu Ulusoy, Serdar Acar, Isa Karaca, Orhan Akpınar, Ozlem Ozmen

**Affiliations:** 1 Department of General Surgery, Faculty of Medicine, Suleyman Demirel University, Isparta, Türkiye; 2 Department of Pharmacology, Faculty of Medicine, Suleyman Demirel University, Isparta, Türkiye; 3 Department of Medical Genetics, Faculty of Medicine, Suleyman Demirel University, Isparta, Türkiye; 4 Department of Electronics and Automation, Isparta University of Applied Sciences, Isparta, Türkiye; 5 Department of Medical Microbiology, Institute of Health Sciences, Suleyman Demirel University, Isparta, Türkiye; 6 Department of Pathology, Faculty of Veterinary Medicine, Burdur Mehmet Akif Ersoy University, Burdur, Türkiye

**Keywords:** Pulsed magnetic field, Radiofrequency electromagnetic field, Liver injury, Oxidative stress, Inflammation, Apoptosis

## Abstract

**Background:**

Sepsis-associated liver injury is a major cause of morbidity and mortality, with oxidative stress, inflammation, and apoptotic signaling playing central roles in pathogenesis. Non-invasive physical modalities such as pulsed magnetic fields (PMF) and radiofrequency electromagnetic fields (RF-EMF) have shown organ-protective effects in various experimental settings; however, their combined application in hepatic inflammation has not been previously investigated.

**Methods:**

Forty female Wistar rats were randomized into five groups: Control, LPS, LPS + PMF, LPS + RF, and LPS + PMF+RF. Acute liver injury was induced with intraperitoneal LPS. PMF and RF-EMF were applied individually or in combination. Liver tissues were analyzed by histopathology, immunohistochemistry, and RT-qPCR for oxidative (NRF2, SOD), inflammatory (TNF-α), mitochondrial apoptotic (BCL2, BAX, Cyt-C, Caspase-9), and ER stress (PERK, Caspase-12, Caspase-3) markers. Serum ALT, AST, and albumin levels were also measured.

**Results:**

LPS significantly increased TNF-α, BAX, Cyt-C, Caspase-9, PERK, Caspase-12, and Caspase-3 expression, while decreasing NRF2, SOD, and BCL2 (all ***p < 0.001). Both PMF and RF monotherapies partially restored these parameters; however, the combined PMF + RF application achieved the most pronounced effects at the molecular and histopathological levels, normalizing oxidative stress markers, reducing pro-inflammatory and apoptotic signaling, and improving histopathological scores (all **p < 0.05 to ***p < 0.001 vs. LPS). Serum AST and ALT levels were significantly reduced by PMF monotherapy, while RF and combined PMF + RF treatments also produced significant decreases compared to the LPS group, albeit to a lesser extent. Serum albumin levels remained unchanged across all groups.

**Conclusion:**

Concurrent low-frequency PMF and RF-EMF exposure confers synergistic hepatoprotection in endotoxin-induced liver injury by modulating oxidative, inflammatory, and apoptotic pathways. These findings suggest that dual-modality PMF and RF-EMF exposure may represent a promising non-invasive experimental strategy for mitigating endotoxin-driven hepatic injury. Further studies in clinically relevant sepsis and ischemia-reperfusion models, with extended follow-up and mechanistic validation, are warranted before translational inference.

**Supplementary Information:**

The online version contains supplementary material available at 10.1007/s00068-026-03119-2.

## Introduction

Sepsis-associated liver injury represents a critical determinant of multi-organ failure, with inflammation, oxidative stress, and mitochondrial dysfunction being central mechanisms driving tissue damage. Lipopolysaccharide (LPS), a component of the Gram-negative bacterial cell wall, is widely employed to simulate systemic inflammation and acute hepatic injury in experimental models due to its robust activation of pro-inflammatory cytokines, reactive oxygen species (ROS), and apoptosis-related pathways [1].

The liver, being a highly vascular and metabolically active organ, is particularly vulnerable to LPS-induced insults. Previous studies have demonstrated that LPS suppresses antioxidant systems, particularly nuclear factor erythroid 2–related factor 2 (NRF2) while upregulating pro-inflammatory mediators such as TNF-α and IL-6 [1, 2]. Additionally, mitochondrial and endoplasmic reticulum (ER) stress responses, marked by increased Caspase-9, Caspase-12, and PERK activation, contribute to apoptotic progression in hepatic tissue [3].

In recent years, biophysical therapeutic approaches such as pulsed magnetic fields (PMF) and radiofrequency electromagnetic fields (RF-EMF) have emerged as promising non-pharmacological modalities to modulate tissue responses. PMF has been shown to influence mitochondrial bioenergetics and redox homeostasis, while RF-EMF is reported to enhance cellular repair and vasodilation through nitric oxide-mediated pathways [4–7].

However, limited data exist regarding their combined application and systemic effects on inflammatory hepatic injury models.

Although PMF and/or RF-EMF have been explored in experimental sepsis-related injury models, the existing literature is largely organ- and axis-specific, typically focusing on a limited set of endpoints (e.g., systemic cytokines/mortality or a single apoptosis pathway) and rarely integrating oxidative defense, mitochondrial stress, ER stress, histopathology, and functional biochemistry within the same hepatic model [8–10]. Moreover, previous studies have typically evaluated these modalities within specific organs or along isolated biological pathways, rather than through an integrated, multi-system analysis within a single experimental model [8–12]. Consequently, the combined effects of low-frequency PMF and RF-EMF on oxidative defense, mitochondrial stress, endoplasmic reticulum stress, histopathological injury, and functional biochemical outcomes within the same endotoxin-induced hepatic model remain insufficiently characterized.

This study was designed to investigate the individual and synergistic therapeutic potential of low-frequency PMF and RF-EMF in LPS-induced acute liver injury. By evaluating key molecular markers related to oxidative stress, inflammation, mitochondrial and ER stress, and apoptosis, this research aims to elucidate the underlying protective mechanisms and support the development of non-invasive strategies for liver protection.

## Materials and methods

### Animals and experimental design

This study included forty adult female Wistar albino rats (250–300 g) obtained from the Experimental Animal Research Unit, Süleyman Demirel University. Animals were housed under standard conditions (21–22 °C, 55–65% humidity, 12-h light/dark cycle) with ad libitum access to standard chow and water. They were acclimatized for one week before any experimental procedures.

Following acclimatization, animals were randomly allocated into five experimental groups (*n* = 8 per group):

1. **Control group**: Rats received an intraperitoneal injection of 1 mL sterile saline to mimic procedural stress and were maintained in standard cages without electromagnetic field exposure for six hours.
2. **LPS group**: Acute systemic inflammation was induced by a single intraperitoneal injection of lipopolysaccharide (LPS, 5 mg/kg). Animals were then kept in conventional cages for six hours without additional intervention.
3. **LPS + PMF group**: Following LPS administration (5 mg/kg, i.p.), rats were exposed to a low-frequency pulsed magnetic field (PMF, 0.5 mT) for three hours using cage-integrated generators and subsequently returned to standard cages for the remaining observation period.
4. **LPS + RF group**: Rats received LPS (5 mg/kg, i.p.) and were exposed to radiofrequency electromagnetic fields (RF-EMF, 27.12 MHz) via embedded circuit board antennas for 30 min, after which they were housed in standard cages for the remaining 5.5 h.
5. **LPS + PMF + RF group**: Animals received LPS (5 mg/kg, i.p.) and were simultaneously exposed to PMF (0.5 mT for 3 h) and RF-EMF (27.12 MHz for 30 min). Following completion of combined exposure, rats were maintained in standard cages until the end of the six-hour experimental period.

A photographic view of the experimental setup is provided in Figure S1, while a schematic illustration of the stimulation configuration is shown in Figure S2 to facilitate reproducibility of the electromagnetic field exposure.

At the end of the six-hour experimental period, animals were anesthetized by intraperitoneal administration of ketamine hydrochloride (90 mg/kg) and xylazine hydrochloride (10 mg/kg). After confirmation of deep anesthesia, euthanasia was performed by surgical exsanguination via the inferior vena cava. Liver tissues were promptly excised; one portion was fixed in 10% neutral-buffered formalin for histopathological and immunohistochemical analyses, while the remaining tissue was snap-frozen in liquid nitrogen and stored at − 80 °C for molecular analyses, including real-time quantitative polymerase chain reaction (RT-qPCR).

### Rationale for PMF and RF-EMF parameters

The PMF and RF-EMF exposure parameters used in this study were selected based on previously published in vivo experimental studies demonstrating biological efficacy without tissue toxicity [10, 11]. A low-intensity PMF (0.5 mT) was chosen as it has been reported to exert anti-inflammatory and cytoprotective effects, particularly through modulation of mitochondrial stability and redox-sensitive pathways, while remaining within a biologically safe exposure window. The RF-EMF frequency of 27.12 MHz corresponds to an internationally accepted industrial, scientific, and medical (ISM) band and has been applied in experimental models to induce non-thermal biological effects, including modulation of vascular and cellular signaling. The combined application was designed to explore whether simultaneous modulation of complementary cellular stress pathways could enhance hepatoprotective efficacy under endotoxin-induced inflammatory conditions. The exposure durations for PMF and RF-EMF were selected according to previously validated experimental protocols, reflecting differences in field characteristics and biological interaction profiles rather than an intention to equalize treatment time [8, 10, 11]. Exposure intensities were intentionally selected within biologically active but non-toxic ranges to allow evaluation of additive or synergistic effects, rather than overwhelming single-modality responses.

### Blood collection and biochemical analysis

At the end of the experimental period, blood samples were collected from the inferior vena cava under deep anesthesia and transferred into serum separator tubes. Samples were allowed to clot at room temperature and subsequently centrifuged at 3000 rpm for 10 min to obtain serum. Serum levels of alanine aminotransferase (ALT), aspartate aminotransferase (AST), and albumin were measured using an automated biochemical analyzer at the Süleyman Demirel University Faculty of Medicine Biochemistry Laboratory, according to standard clinical protocols and manufacturer instructions.

Liver tissues obtained for oxidative stress analyses were portioned, placed in Eppendorf tubes, and stored at − 80 °C until analysis. Tissues were homogenized in phosphate-buffered saline (10 mM sodium phosphate, pH 7.4) at a ratio of 1:5 (w/v) using a tissue homogenizer (IKA Ultra Turrax T25, Janke & Kunkel, Staufen, Germany). Homogenates were centrifuged at 2000 rpm for 20 min at 4 °C (Nuve NF 1200R, Ankara, Türkiye), and the supernatants were transferred to the Süleyman Demirel University Faculty of Medicine Biochemistry Laboratory for triplicate measurements. Oxidative stress status was evaluated by measuring total antioxidant status (TAS) and total oxidant status (TOS) using the spectrophotometric method described by Erel [13, 14]. TOS results were expressed as µmol H₂O₂ equivalent per gram protein, and TAS results as mmol Trolox equivalent per gram protein. The oxidative stress index (OSI) was calculated as the ratio of TOS to TAS (OSI = TOS/TAS/10).

### Histopathological analysis

Liver samples were collected at necropsy and fixed in 10% neutral-buffered formalin. Following routine tissue processing, specimens were embedded in paraffin, and 5-µm sections were obtained using a rotary microtome. Sections were stained with hematoxylin and eosin (H&E) and examined under a light microscope for histopathological evaluation. Hepatic injury was assessed based on the presence and severity of necrosis, inflammation, and hemorrhage, as summarized in Table 1.

**Table 1:** Histopathology score of hepatic lesions

Histological criteria	Severity	Description	Score
Hemorrhage	Absent		0
	Mild	< 3 foci	1
	Marked	4–6 foci	2
	Severe	> 7 foci	3
Inflammation	None		0
	Moderate	Scattered	1
	Marked	Foci	2
	Severe	Diffuse	3
Necrosis	Absent	0%	0
	Mild	< 10%	1
	Marked	10–50%	2
	Severe	> 50%	3

### Immunohistochemical examination

Three serial sections were obtained from each paraffin block and mounted on poly-L-lysine–coated slides for immunohistochemical analysis. Immunoreactivity for Caspase-3 (EPR18297; ab184787), superoxide dismutase-1 (SOD-1; EP1727Y; ab51254), and cytochrome C (EPR1327; ab133504) was evaluated using a streptavidin–biotin peroxidase–based method. Primary antibodies (Abcam, Cambridge, UK) were applied at a dilution of 1:100 and incubated for 60 min. Sections were subsequently treated with a biotinylated secondary antibody, followed by streptavidin–horseradish peroxidase, and immunoreactivity was visualized using 3,3′-diaminobenzidine (DAB) with the Abcam Mouse and Rabbit Specific HRP/DAB IHC Detection Kit (ab236466).

Negative controls were prepared by substituting the primary antibodies with antibody diluent. All immunohistochemical evaluations were performed by an experienced pathologist from an independent institution, blinded to group allocation. The percentage of positively stained cells for each marker was assessed at ×40 magnification by examining ten randomly selected, non-overlapping high-power fields per section, chosen from representative areas of the hepatic parenchyma while avoiding large vessels, bile ducts, and tissue artifacts. Each field corresponded to a standardized microscopic area. Quantitative analysis was conducted using ImageJ software (National Institutes of Health, Bethesda, MD; version 1.48), and representative microphotographs were captured using the Database Manual Cell Sens Life Science Imaging Software System (Olympus Co., Tokyo, Japan).

### Reverse transcription–quantitative polymerase chain reaction (RT-qPCR)

Total RNA was extracted from homogenized liver tissues using the GeneAll RiboEx™ RNA Isolation Kit (GeneAll Biotechnology, Seoul, Korea), according to the manufacturer’s instructions. RNA concentration and purity were assessed using a BioSpec-nano spectrophotometer (Shimadzu, Kyoto, Japan). For complementary DNA (cDNA) synthesis, 1 µg of total RNA was reverse-transcribed using the A.B.T.™ cDNA Synthesis Kit (Atlas Biotechnology, Türkiye) in a thermal cycler following the manufacturer’s protocol.

Primer sequences were designed based on target mRNA sequences using the NCBI database, and the primer sequences used in the study are presented in Table 2. Quantitative real-time PCR was performed using a Bio-Rad CFX96 Real-Time PCR System (Bio-Rad, USA) with 2× SYBR Green Master Mix (Nepenthe, Türkiye). GAPDH was used as the housekeeping gene for normalization. Each reaction was prepared in a final volume of 20 µL and run in triplicate.

**Table 2:** Primary sequences, product size and accession numbers of genes

Genes	Primary sequence	product size	accession number
GAPDH (HouseKeeping)	F: AGTGCCAGCCTCGTCTCATA	248 bp	NM_017008.4
	R: GATGGTGATGGGTTTCCCGT		
Bcl-2	F: CATCTCATGCCAAGGGGGAA	284 bp	NM_016993.2
	R: TATCCCACTCGTAGCCCCTC		
Bax	F. CACGTCTGCGGGGAGTCAC	419 bp	NM_017059.2
	R: TAGAAAAGGGCAACCACCCG		
Cas9	F: AGCCAGATGCTGTCCCATAC	148 bp	XM_039110693.1
	R: CAGGAACCGCTCTTCTTGTC		
Cas12	F: CTGCATCAGAATCCAGGGGA	212 bp	NM_130422.1
	R: TCGGCCTTCCTTCTCCATCA		
Perk	F: CCAAGCTGTACATGAGCCCAGA	178 bp	XM_008762977.4
	R: TTCTGAGTGAACAGTGGTGGAAAC		
TNF α	F: TCGTAGCAAACCACCAAGCA	208 bp	NM_012675.3
	R: GAAGTGGCAAATCGGCTGAC		
NRF2	F: GCCTTCCTCTGCTGCCATTAGTC	126 bp	NM_001399173.1
	R: TCATTGAACTCCACCGTGCCTTC		

F: Forward, R: Reverse, GAPDH: Glyceraldehyde-3-phosphate dehydrogenase, Bcl-2: B-cell lymphoma 2, Bax: BCL2-associated X, Cas-9: Caspase-9, Cas-12: Caspase-12, PERK: PRKR-like endoplasmic reticulum kinase, TNF α: Tumor Necrosis Factor alpha NRF2: Nuclear factor erythroid 2-like 2

The RT-qPCR cycling conditions consisted of an initial denaturation at 94 °C for 10 min, followed by 45 cycles of denaturation at 95 °C for 15 s and annealing/extension at 57 °C for 30 s. Relative mRNA expression levels were calculated using the 2^−ΔΔCt method after normalization to the housekeeping gene GAPDH and expressed relative to the control group.

### Statistical analysis

Statistical analysis was performed using GraphPad Prism. The Shapiro-Wilk test was initially applied to assess the normality of the data distribution. As the data exhibited a normal distribution (*p* > 0.05), a one-way analysis of variance (ANOVA) was used to compare the groups. To identify pairwise differences between the groups, the post hoc Tukey test was employed. The results are presented as means ± standard deviations, with a significance level of *p* < 0.05.

### Ethical approval

The experiment was conducted in accordance with the Animal Research: Reporting of In Vivo Experiments (ARRIVE) 2.0 guidelines. Ethical approval was obtained from the Süleyman Demirel University Animal Experiments Local Ethics Committee (SDÜ-HADYEK; Approval No: 12.06.2025-06/528). The study was financially supported by the Süleyman Demirel University Scientific Research Project Unit (SDU-BAP) (Project No: TSG-2024-9515).

## Results

### Inflammatory panel (TNF-α, histopathological inflammation scores)

**Fig. 1:**
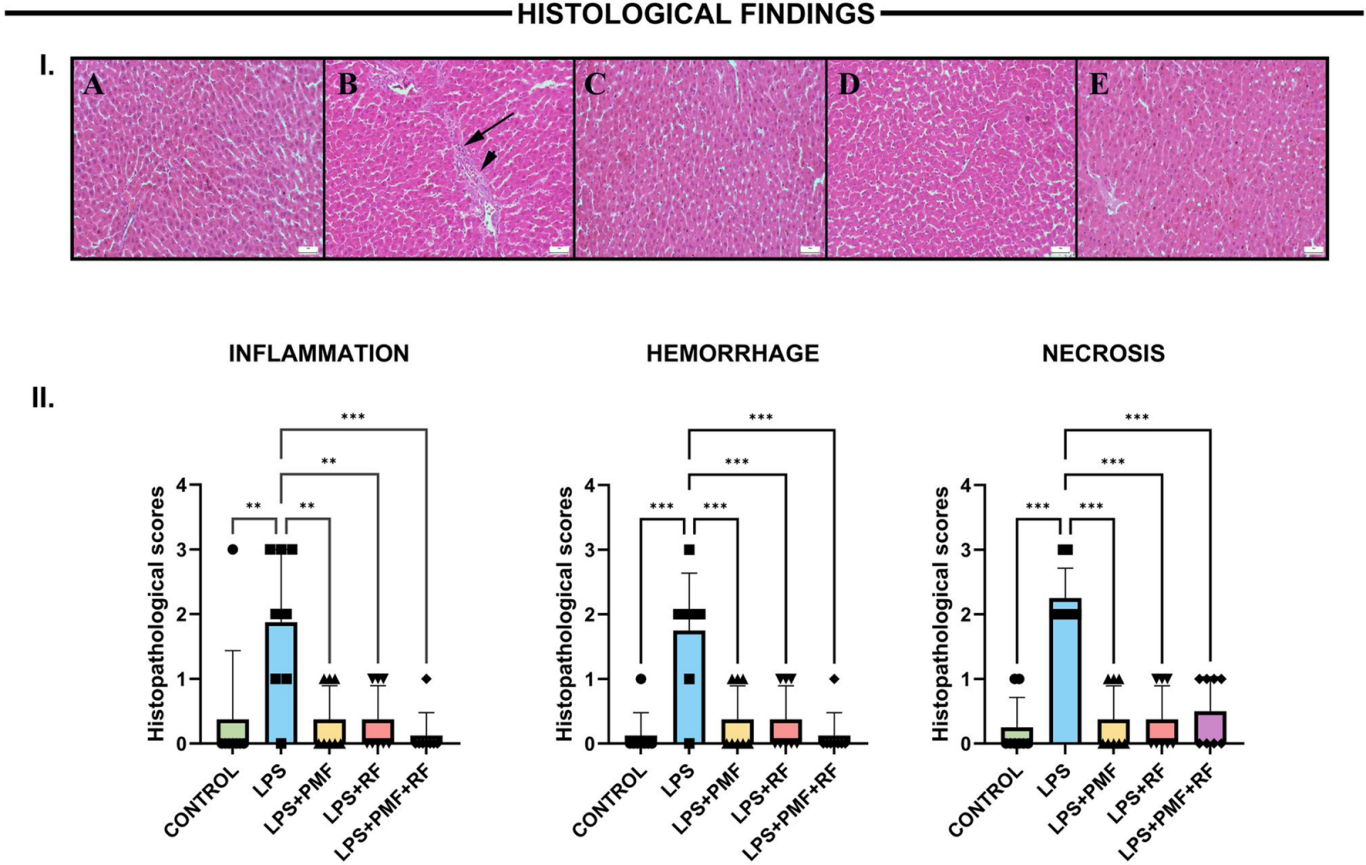
Histopathological effects of pulsed magnetic field (PMF) and radiofrequency electromagnetic field (RF) applications on liver inflammation following LPS-induced injury. (I) **(A)** Normal liver architecture in the control group. (**B**) The LPS group exhibits pronounced hyperemia (arrowhead), hemorrhage, inflammatory cell infiltration (arrow), and hepatocyte degeneration. (**C**) Reduced pathological alterations in the PMF group. (**D**) Decreased pathological alterations in the RF group. (**E**) Normal liver histology observed in the PMF + RF group. HE staining; scale bars = 50 μm. **(II)** Histopathological evaluation showed significantly higher scores for inflammation, hemorrhage, and necrosis in the LPS group, which were markedly ameliorated in all treatment groups, particularly in LPS + PMF+RF (**p < 0.01 to ***p < 0.001)

Histological scoring corroborated the molecular findings. The LPS group exhibited severe inflammation, prominent hemorrhagic areas, and widespread necrosis, with significantly elevated scores across all parameters (***p < 0.001 vs. control). In contrast, animals receiving electromagnetic-based interventions displayed considerable histological improvements. The LPS + PMF+RF group demonstrated the most substantial amelioration, with all pathological scores significantly reduced (***p < 0.001 vs. LPS) (Fig. 1). These improvements were visibly evident in the corresponding micrographs, where inflammatory cell infiltration and hemorrhagic foci were notably diminished. Together, these findings indicate that combined PMF and RF therapy exert robust anti-inflammatory effects, potentially by suppressing TNF-α-driven signaling cascades and mitigating histological damage in hepatic tissue following septic challenge.

**Fig. 2:**
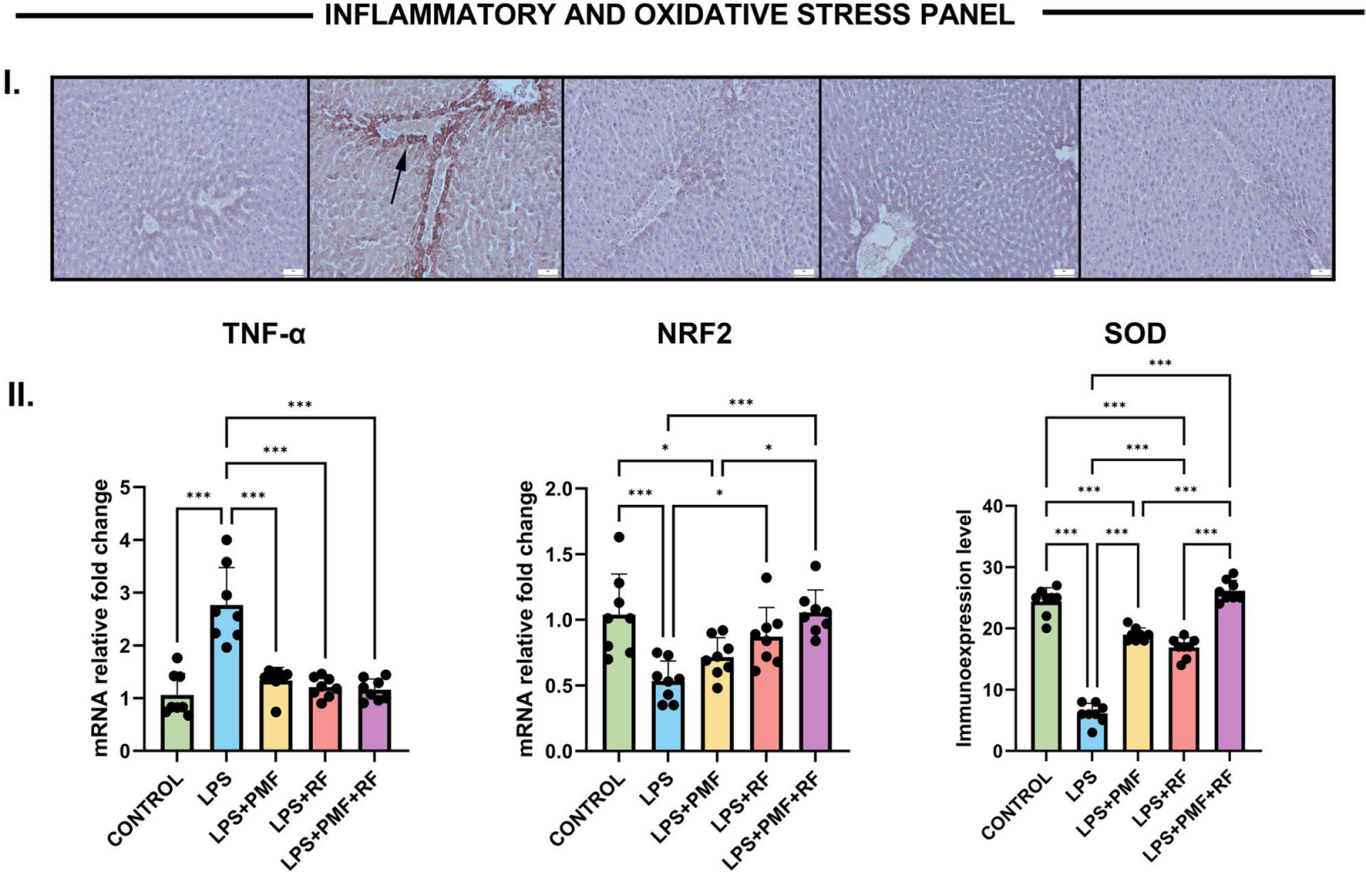
Effects of PMF and RF on inflammation related TNF-α and oxidative stress-related NRF2 and SOD levels in liver tissue following LPS-induced injury. I. Immunohistochemical expression of SOD in liver tissues across different groups. (**A**) Negative expressions in the control group. (**B**) Marked elevation in hepatocytes (arrows) in the LPS group. (**C**) Reduced expression levels in the PMF group. (**D**) Decreased expressions in the RF group. (**E**) Slight to negative expression in the PMF + RF group. Streptavidin-biotin peroxidase method, scale bars = 50 μm. **II.** Quantitative analysis of TNF-α, and NRF2 gene expression and SOD protein immunoexpression in liver tissue. Data are presented as mean ± SEM. A significant upregulation of TNF-α and downregulation of NRF2 mRNA expression was observed in the LPS group compared to control (***p < 0.001 for both), while PMF and/or RF treatments partially restored expression levels, with the combined LPS + PMF+RF group showing the highest recovery (*p < 0.05 to ***p < 0.001). Similarly, SOD immunoreactivity was significantly reduced in the LPS group compared to the control (***p < 0.001), and notably restored in the LPS + PMF+RF group (***p < 0.001 vs. LPS), indicating a synergistic antioxidative effect of dual-modality treatment. The increased immunoreactivity observed in the LPS group likely reflects a stress-induced compensatory response rather than enhanced functional antioxidant activity, which is consistent with the reduced NRF2 and SOD levels detected by biochemical and molecular analyses

To evaluate the anti-inflammatory efficacy of PMF and RF applications, TNF-α gene expression and histopathological scoring for inflammation, hemorrhage, and necrosis were analyzed in liver tissues subjected to LPS-induced injury. TNF-α mRNA levels were markedly increased in the LPS group compared to controls (***p < 0.001), highlighting a strong pro-inflammatory response. Both PMF and RF monotherapies significantly reduced TNF-α expression (***p < 0.001 vs. LPS for both), whereas the combination of PMF and RF provided the most pronounced inhibitory effect, restoring TNF-α levels toward baseline (***p < 0.001 vs. LPS) (Fig. 2).

### Oxidative stress panel (NRF2 & SOD)

The expression levels of key oxidative stress-related markers were evaluated to determine the protective efficacy of PMF and RF applications (Fig. 2). In the NRF2 panel, the LPS group demonstrated a statistically significant suppression in NRF2 mRNA expression compared to the control group (***p < 0.001), confirming oxidative imbalance. Administration of RF as individual therapy partially increased NRF2 expression (*p* < 0.05 vs. LPS), while the combined application (LPS + PMF+RF) yielded a more pronounced effect, restoring levels closer to baseline (***p < 0.001 vs. LPS).

Parallel immunohistochemical analysis of SOD protein expression revealed a consistent trend (Fig. 2). SOD levels were markedly reduced following LPS exposure (***p < 0.001), while co-treatment with PMF and RF significantly reversed this suppression (***p < 0.001 vs. LPS), suggesting a strong antioxidative synergy. This was supported by histological images, where SOD-positive immunostaining appeared diminished in the LPS group and visibly recovered in the LPS + PMF+RF group. These findings indicate that dual-modality electromagnetic treatment effectively restores antioxidant defense mechanisms in LPS-induced hepatic oxidative injury.

### Mitochondrial stress panel (BCL2, BAX, Cyt-C, CAS-9)

**Fig. 3:**
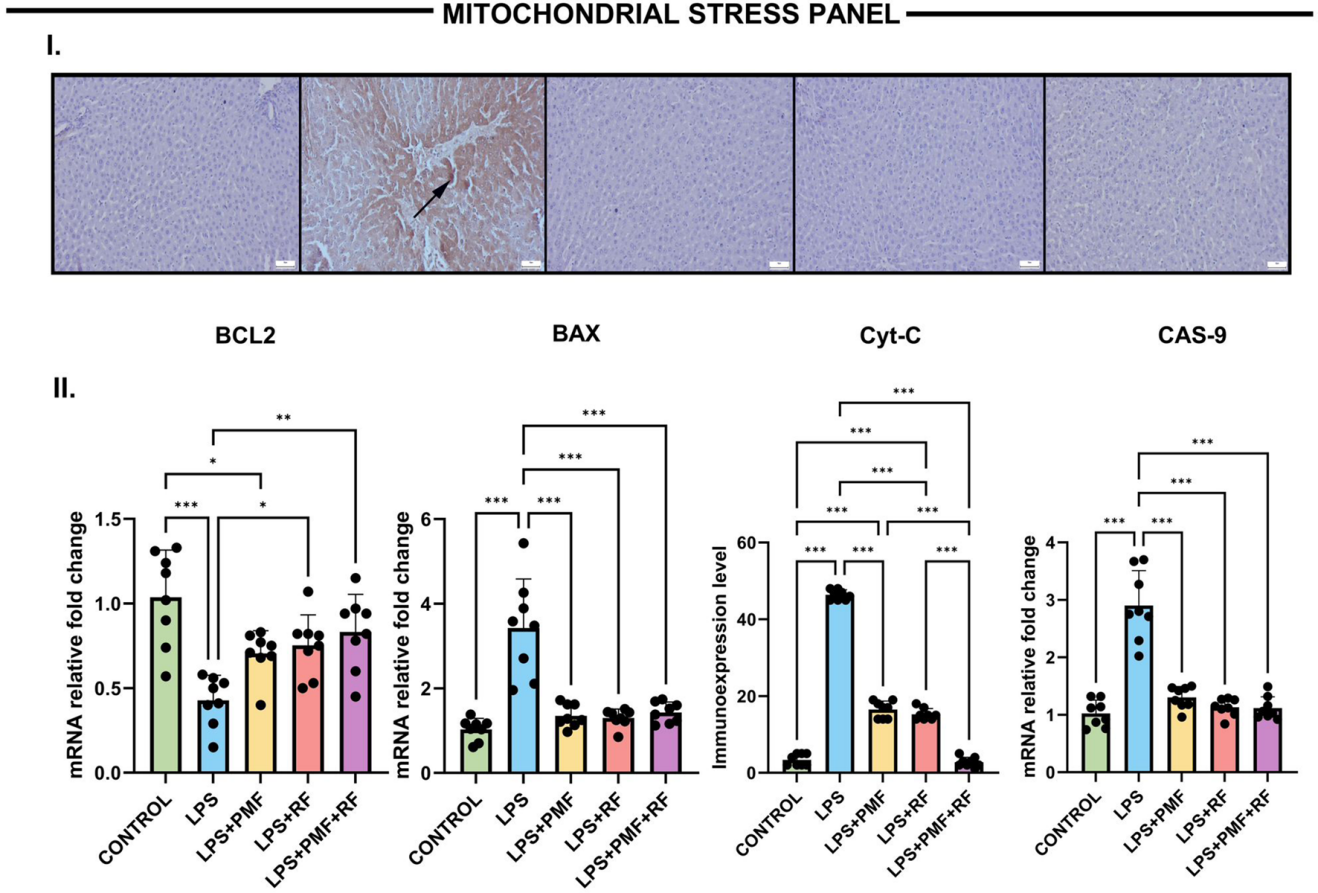
Effects of PMF and RF treatments on mitochondrial apoptosis-related gene and protein expression in LPS-induced liver injury. I. (**A**) Immunohistochemical expression of Cytochrome C (Cyt-C) in liver tissues across different groups. (**A**) Negative expressions in the control group. (**B**) Marked elevation in hepatocytes (arrows) in the LPS group. (**C**) Reduced expression levels in the PMF group. (**D**) Decreased expressions in the RF group. (**E**) Slight to negative expression in the PMF + RF group, Streptavidin-biotin peroxidase method, scale bars = 50 μm. **II.** Effects of PMF and RF treatments on mitochondrial apoptosis-related gene and protein expression in LPS-induced liver injury. Cyt-C IHC scores and quantitative analysis of mitochondrial stress markers: BCL2, BAX, and Caspase-9 (CAS-9). LPS administration significantly downregulated anti-apoptotic BCL2 expression while upregulating pro-apoptotic BAX and CAS-9 mRNA levels and Cyt-C protein immunoreactivity (***p < 0.001). Both PMF and RF interventions mitigated these mitochondrial apoptotic responses, with the combined treatment (LPS + PMF+RF) showing the most robust reversal (*p < 0.05 to ***p < 0.001)

To explore the potential mitochondrial-mediated apoptotic effects of LPS and the protective impact of electromagnetic therapies, we quantified the expression of key regulators involved in intrinsic apoptosis. As shown in Fig. 3, the LPS group exhibited a significant reduction in the anti-apoptotic gene BCL2 (**p < 0.001 vs. control), alongside marked elevations in pro-apoptotic markers BAX and CAS-9 (***p < 0.001 for both). These findings are consistent with LPS-induced mitochondrial dysfunction and apoptotic activation.

PMF and RF monotherapies resulted in partial normalization of these expression levels. Notably, the combined LPS + PMF+RF group demonstrated the most substantial therapeutic effect, with BCL2 levels approaching those of the control group (**p < 0.01 vs. LPS) and a significant reduction in BAX and CAS-9 mRNA expression (***p < 0.001 vs. LPS for both). Furthermore, immunohistochemical staining for Cyt-C revealed intense cytoplasmic immunoreactivity in the LPS group, indicative of mitochondrial outer membrane permeabilization and apoptosome formation. This was markedly reduced in all treatment groups, particularly in LPS + PMF+RF, highlighting the protective effect of dual-modality therapy in preventing mitochondrial apoptotic signaling.

These results suggest that electromagnetic field applications not only prevent LPS-induced oxidative damage but also modulate mitochondrial apoptotic pathways by restoring the BCL2/BAX balance, suppressing Cyt-C release, and inhibiting Caspase-9 activation—key steps in intrinsic apoptotic signaling.

### ER stress and apoptosis panel (PERK, CAS-12, CAS-3)

**Fig. 4:**
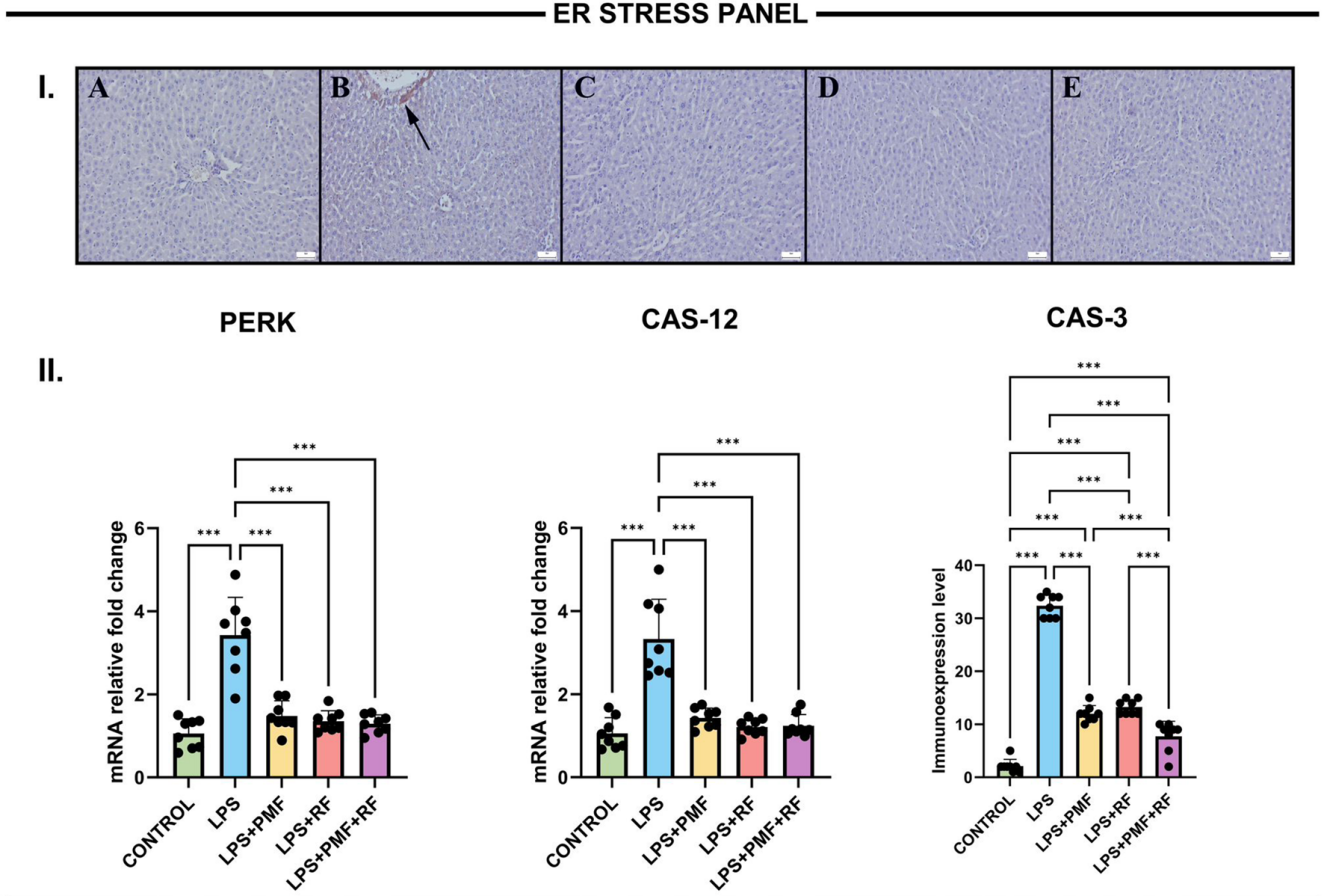
Effects of PMF and RF on endoplasmic reticulum (ER) stress and apoptosis markers in LPS-induced liver injury. I. Immunohistochemical expression of Caspase-3 in liver tissues across different groups. (**A**) Negative in the control group. (**B**) Marked elevation in hepatocytes (arrows) in the LPS group. (**C**) Reduced expression levels in the PMF group. (**D**) Decreased expressions in the RF group. (**E**) Slight to negative expression in the PMF + RF group. The streptavidin-biotin peroxidase method was used; scale bars = 50 μm. **II.** Quantitative analysis of ER stress and apoptosis-related markers: PERK and CAS-12 mRNA expression, and Caspase-3 (CAS-3) protein immunoreactivity. LPS administration significantly increased PERK and CAS-12 gene expression levels, as well as CAS-3 protein expression (***p < 0.001 vs. control), suggesting activation of the unfolded protein response and ER stress-induced apoptosis. Treatments with PMF and RF significantly reduced these markers, with the combined PMF + RF treatment producing the most robust suppression (***p < 0.001)

To evaluate ER stress and related apoptotic activation, PERK, CAS-12, and CAS-3 levels were assessed in liver tissues. As shown in Fig. 4, the LPS group demonstrated a significant increase in PERK mRNA expression (***p < 0.001), reflecting the activation of the PERK arm of the unfolded protein response (UPR). This upregulation was significantly attenuated in all treatment groups, with the most notable suppression observed in the LPS + PMF+RF group (***p < 0.001 vs. LPS), suggesting synergistic inhibition of ER stress signaling.

Similarly, CAS-12 expression, a specific marker of ER stress-induced apoptosis, was markedly elevated in the LPS group (***p < 0.001), confirming activation of ER stress-related apoptotic pathways. PMF and RF treatments significantly reduced CAS-12 expression levels, again with the dual treatment providing the strongest protective effect.

Immunohistochemical analysis of CAS-3 (Fig. 4) further supported these findings. While the LPS group showed intense CAS-3 immunopositivity, indicative of downstream apoptotic execution, treated groups displayed significantly decreased staining, reflecting effective mitigation of apoptotic processes via modulation of both ER and mitochondrial stress axes.

These results underscore the potential of combined pulsed magnetic field and radiofrequency electromagnetic field applications in counteracting ER stress-mediated apoptosis, likely through downregulation of the PERK/eIF2α and CAS-12 pathways, ultimately preventing activation of the apoptotic executor CAS-3.

### Blood biochemistry panel (AST, ALT, Albumin)

**Fig. 5:**
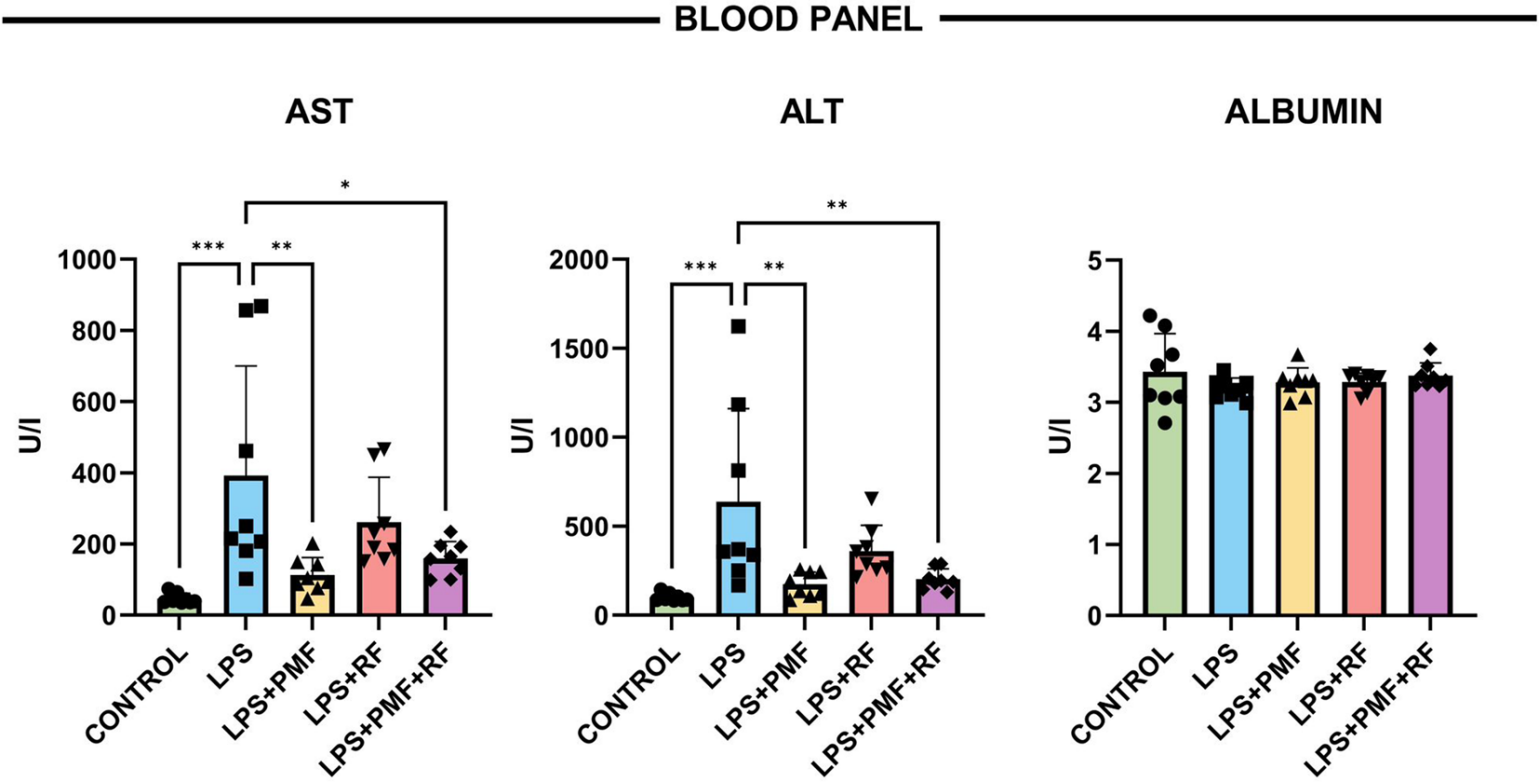
Effects of PMF and RF applications on serum liver function parameters in LPS-induced hepatic injury. Serum levels of aspartate aminotransferase (AST), alanine aminotransferase (ALT), and albumin were measured to assess biochemical indicators of hepatic injury. LPS exposure significantly elevated AST and ALT levels compared to the control group (***p < 0.001). PMF monotherapy resulted in the most pronounced reduction in serum transaminase levels, while RF and combined PMF + RF treatments also produced significant but comparatively less marked decreases (*p < 0.05 to **p < 0.01 vs. LPS). Serum albumin levels remained stable across all experimental groups, with no statistically significant differences observed

To investigate the systemic biochemical indicators of hepatic injury and the therapeutic efficacy of electromagnetic interventions, serum levels of AST, ALT, and albumin were quantified. As shown in Fig. 5, both AST and ALT were significantly elevated in the LPS group compared to controls (***p < 0.001 for both), reflecting substantial hepatocyte membrane damage and necrotic leakage of intracellular enzymes, hallmark features of LPS-induced hepatotoxicity.

Application of PMF and RF therapies resulted in significant reductions in AST and ALT levels compared to the LPS group. Notably, PMF monotherapy produced the most pronounced reduction in serum transaminases, whereas the combined PMF + RF treatment also significantly lowered enzyme levels (*p < 0.05 to **p < 0.01), although to a lesser extent than PMF alone.

Serum albumin concentrations remained within the normal range across all experimental groups, with no significant intergroup differences, indicating preserved hepatic synthetic function and supporting the specificity of AST and ALT elevations as markers of hepatocellular injury rather than global liver failure.

## Discussion

This study provides robust evidence that PMF and RF-EMF applications, particularly when combined, exert significant hepatoprotective effects in a rat model of LPS-induced liver injury. The findings demonstrate that these physical modalities modulate multiple pathophysiological axes, including oxidative stress, inflammation, mitochondrial and ER stress, and apoptosis. Notably, the combined PMF + RF application yielded superior protective outcomes compared to either modality alone, suggesting a synergistic interaction.

Prior experimental work has reported protective effects of electromagnetic modalities in sepsis-related injury models; however, the scope of endpoints and the target organ vary substantially across studies. Previous investigations have often focused on a single modality (PMF/PEMF or RF-EMF) and a restricted mechanistic axis such as cytokine output or an apoptosis-hypoxia signature, or they have addressed safety rather than therapeutic efficacy in hepatic tissue [8–12]. In contrast, our study evaluates concurrent low-frequency PMF and 27.12 MHz RF-EMF within the same endotoxin-driven hepatic injury model and integrates inflammatory signaling (TNF-α), oxidative defense (NRF2/SOD), mitochondrial apoptosis (BCL2/BAX/CAS-9/Cyt-C), ER stress–related apoptosis (PERK/CAS-12/CAS-3), histopathological damage scores, and serum biochemistry (AST/ALT). This multi-domain design helps delineate how different stress compartments in the liver respond in parallel to single versus combined electromagnetic exposures, thereby extending the current organ- and endpoint-limited literature.

LPS-induced hepatic injury is driven by a cascade of oxidative insults, with suppressed antioxidant defense systems such as NRF2 playing a central role in tissue vulnerability [1].

In this study, NRF2 mRNA expression was significantly downregulated following LPS exposure, consistent with previous literature linking LPS to impaired redox homeostasis and reduced nuclear translocation of NRF2. Treatment with PMF and RF, especially in combination, effectively restored NRF2 expression and SOD immunoreactivity, indicating reactivation of the cellular antioxidant machinery. These findings align with studies showing that PMF can enhance ROS scavenging and modulate redox-sensitive transcription factors, while RF has been linked to redox modulation through cellular membrane potential stabilization and ionic flux regulation [15].

The inflammatory response was another critical target of PMF and RF intervention. TNF-α, a central mediator in LPS-induced cytokine storms, was markedly upregulated in the LPS group. Its suppression by electromagnetic therapies demonstrates potent anti-inflammatory effects. PMF has been shown to inhibit TNF-α secretion by macrophages and endothelial cells through IL-6/TLR4 signaling interference. RF, on the other hand, may reduce leukocyte adhesion and endothelial activation, indirectly attenuating cytokine release [16, 17]. In our study, histopathological inflammation, necrosis, and hemorrhage scores mirrored the TNF-α trends, further reinforcing the anti-inflammatory efficacy of electromagnetic exposure.

Mitochondrial apoptotic markers were significantly altered in the LPS group, where increased BAX, CAS-9, and Cyt-C levels, alongside suppressed BCL2 expression, reflected prominent intrinsic apoptosis. This aligns with earlier findings where LPS-induced mitochondrial dysfunction led to membrane depolarization, ROS accumulation, and Cyt-C release. Electromagnetic therapies successfully restored BCL2/BAX homeostasis and reduced CAS-9 and Cyt-C levels, thereby preserving mitochondrial membrane integrity. These effects are likely mediated via PMF’s influence on mitochondrial permeability transition pore (mPTP) regulation and RF’s stabilization of mitochondrial dynamics via the PINK1/Parkin pathway [18–20].

Importantly, this study also highlights the involvement of the ER stress-apoptosis axis in LPS-induced hepatotoxicity. Elevated PERK and CAS-12 gene expression in the LPS group confirmed activation of the UPR and ER-dependent apoptotic signaling. Electromagnetic treatments especially the combined approach resulted in a pronounced downregulation of these markers. Previous reports have shown that PMF can suppress ER stress by reducing GRP78 and CHOP activation, while RF-EMF has been implicated in modulating ER calcium homeostasis, thus preventing protein misfolding-induced apoptosis. The additional decrease in CAS-3 expression common to both mitochondrial and ER stress pathways underscore the global anti-apoptotic effect of PMF + RF treatment [21, 22].

Importantly, the consistent suppression of PERK, CAS-12, and CAS-3 expression observed in the combined PMF + RF group aligns with the marked attenuation of mitochondrial apoptotic signaling, suggesting coordinated modulation of ER-mitochondrial crosstalk rather than isolated pathway inhibition. This integrated response may help explain the superior tissue-level protection observed with combined treatment, despite modest differences in serum transaminase normalization.

Histopathological evaluation provided morphological corroboration of molecular findings. The LPS group showed extensive hepatocellular damage, including cytoplasmic vacuolization, necrosis, and leukocyte infiltration. These features were significantly attenuated in the PMF and RF-treated groups. Notably, the PMF + RF group showed near-complete histological normalization, suggesting effective tissue-level resolution of injury. This aligns with earlier reports of PMF promoting hepatocyte regeneration and RF reducing tissue hypoxia and microvascular injury [6, 9, 23, 24].

In terms of systemic markers, elevated ALT and AST levels in the LPS group confirmed hepatocyte membrane disruption and enzyme leakage into circulation. Both PMF and RF treatments independently improved these biochemical parameters, with the combined treatment achieving near-baseline normalization. The unchanged albumin levels across groups suggest that the synthetic function of the liver was largely preserved during the acute injury window, and the observed enzyme alterations primarily reflect hepatocellular damage rather than hepatic failure.

Although PMF monotherapy was associated with a more pronounced normalization of serum transaminase levels, biochemical improvement alone does not fully reflect the complexity of endotoxin-induced hepatic injury. Notably, while AST and ALT reductions were more prominent in the PMF group, the combined PMF + RF treatment consistently demonstrated broader modulation across multiple injury-related pathways, including oxidative stress balance, inflammatory signaling, mitochondrial apoptotic cascades, and endoplasmic reticulum stress responses. These findings suggest that dual-modality electromagnetic exposure may confer hepatoprotection through coordinated multi-axis regulation rather than isolated biochemical normalization.

The synergistic effect of PMF and RF is a notable highlight of this study. While each modality independently improved markers of injury, their combination produced greater therapeutic efficacy across virtually all measured parameters. This suggests that the mechanisms by which PMF and RF act may be complementary. PMF primarily modulates ion channel conductance and intracellular signal cascades (like MAPK and PI3K/Akt), while RF may influence tissue conductivity, dielectric properties, and local thermal microenvironments, enhancing perfusion and oxygenation [6, 9].

Our findings also carry important translational relevance. Given the non-invasive, low-energy nature of these electromagnetic therapies, they hold promise for clinical application in conditions involving acute hepatic injury, such as drug-induced liver injury, ischemia-reperfusion, or septic shock. The devices used in this study, cage-based coil and antenna systems, can be adapted to human-safe devices such as transdermal applicators or wearable RF-PMF patches. However, further studies are needed to explore long-term safety, repeated-dose effects, and interactions with pharmacologic agents.

There are some limitations in this study. The observation period was limited to six hours post-LPS exposure, focusing on early-phase injury. Longer-term studies are required to evaluate regenerative responses and fibrosis. Additionally, while molecular and histological data support protective effects, deeper pathway analyses (e.g., NF-κB, Nrf2/Keap1, ATF6, IRE1) through Western blotting or transcriptomics would enhance mechanistic clarity.

One of the major challenges limiting the clinical translation of physical stimulation modalities is the lack of standardized exposure protocols and insufficient methodological detail across experimental studies, which complicates reproducibility. In the present study, we aimed to minimize this limitation by providing a detailed description of the PMF and RF-EMF exposure parameters, experimental setup, and outcome assessment methods. Nevertheless, as with most preclinical investigations, protocol heterogeneity across studies remains an inherent barrier to direct clinical extrapolation and warrants cautious interpretation of translational relevance.

From a translational standpoint, although direct clinical application cannot yet be inferred, the findings of this study may carry important implications for future general surgical practice, particularly in the context of postoperative sepsis, ischemia-reperfusion injury, or drug-induced hepatic dysfunction commonly encountered in intensive care settings. The rapid and non-invasive nature of PMF and RF-EMF therapies offers a promising adjunctive strategy to mitigate acute hepatic injury without introducing pharmacological burden. In clinical scenarios such as abdominal sepsis or post-hepatectomy systemic inflammatory response, electromagnetic field-based interventions may help preserve hepatocellular integrity, support antioxidant defense systems, and reduce inflammation-driven cellular apoptosis.

Although translation into clinical practice requires further validation, this pioneering work lays the groundwork for future preclinical and translational studies aiming to explore electromagnetic field-based interventions in hepatic inflammation, sepsis-associated liver dysfunction, and postoperative inflammatory responses.

## Conclusion

In summary, this study demonstrates that low-frequency PMF and RF-EMF therapies, especially when applied concurrently, offer significant protection against LPS-induced liver injury. The dual-modality treatment effectively attenuated oxidative stress, suppressed pro-inflammatory cytokine expression, preserved mitochondrial integrity, and reduced endoplasmic reticulum stress and apoptosis. These findings were corroborated by both molecular and histopathological analyses, alongside improvements in serum liver function markers.

Importantly, the synergistic interaction observed between PMF and RF applications suggests that simultaneous modulation of redox, inflammatory, and apoptotic pathways can provide a multi-dimensional therapeutic benefit in the context of acute hepatic inflammation. As non-invasive, easily applicable modalities, electromagnetic field therapies hold translational potential for future use in clinical settings involving septic liver damage or other inflammatory liver pathologies. Further investigations into long-term effects, repeated dosing, and underlying signaling networks will be critical for advancing their clinical integration.

## Supplementary Information

Below is the link to the electronic supplementary material.

Supplementary Material 1: Fig. S1: Photographic documentation of the experimental electromagnetic field exposure chamber. The image illustrates the custom-built, non-conductive exposure chamber used for in vivo electromagnetic field applications. The radiofrequency electromagnetic field (RF-EMF, 27.12 MHz) applicator is mounted on the upper inner surface of the chamber (red arrow), while the low-frequency pulsed magnetic field (PMF, 0.5 mT) coil system is positioned on the lower surface (yellow arrow). The interior surfaces of the chamber were shielded to minimize external electromagnetic interference and to provide controlled exposure conditions. Animals were placed centrally within the chamber during stimulation without direct contact with the field-generating components, allowing simultaneous RF-EMF and PMF exposure under non-thermal experimental conditions

Supplementary Material 2: Fig. S2: Schematic representation of the experimental setup used for electromagnetic field exposure. Animals were placed centrally within a non-conductive exposure chamber. Low-frequency pulsed magnetic field (PMF, 0.5 mT) was applied from the lower coil system, while radiofrequency electromagnetic field (RF-EMF, 27.12 MHz) exposure was delivered from the upper applicator. The configuration was designed to ensure homogeneous field exposure and to avoid physical contact or thermal effects during stimulation.

## Data Availability

The data that support the findings of this study are available from the corresponding author upon reasonable request.
