# Supplementary figures and images for: Synergistic mitigation of endotoxin-induced liver injury by low-frequency PMF and 27.12 MHz RF-EMF: a multi-biomarker experimental study
Source: Eur J Trauma Emerg Surg. 2026 Feb 23;52(1):56. doi: 10.1007/s00068-026-03119-2 (PMC12929223; doi:10.1007/s00068-026-03119-2)

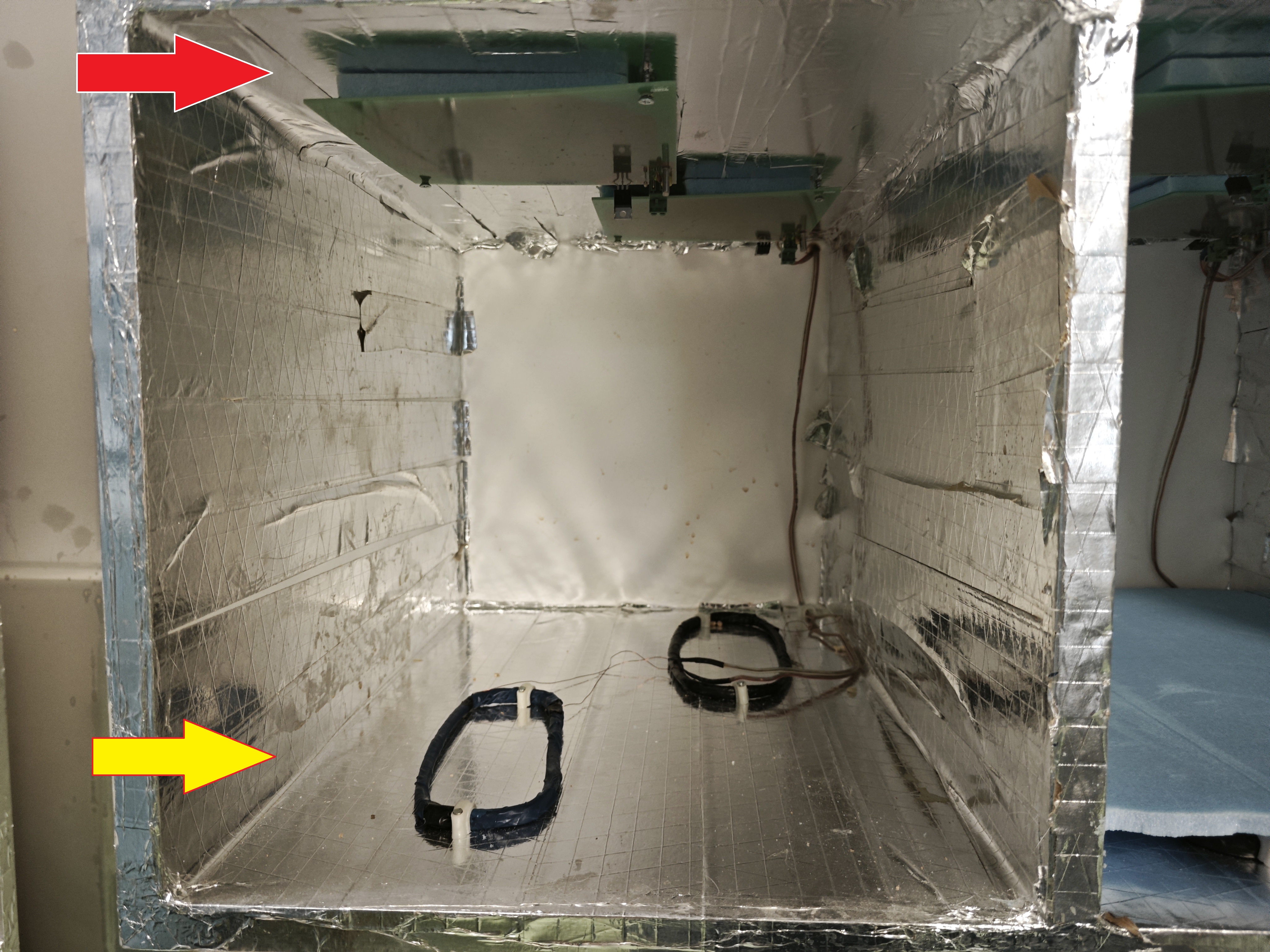

Supplement: Supplementary file 1 — Supplementary Material 1: Fig. S1: Photographic documentation of the experimental electromagnetic field exposure chamber. The image illustrates the custom-built, non-conductive exposure chamber used for in vivo electromagnetic field applications. The radiofrequency electromagnetic field (RF-EMF, 27.12 MHz) applicator is mounted on the upper inner surface of the chamber (red arrow), while the low-frequency pulsed magnetic field (PMF, 0.5 mT) coil system is positioned on the lower surface (yellow arrow). The interior surfaces of the chamber were shielded to minimize external electromagnetic interference and to provide controlled exposure conditions. Animals were placed centrally within the chamber during stimulation without direct contact with the field-generating components, allowing simultaneous RF-EMF and PMF exposure under non-thermal experimental conditions [file 68_2026_3119_MOESM1_ESM.jpg]

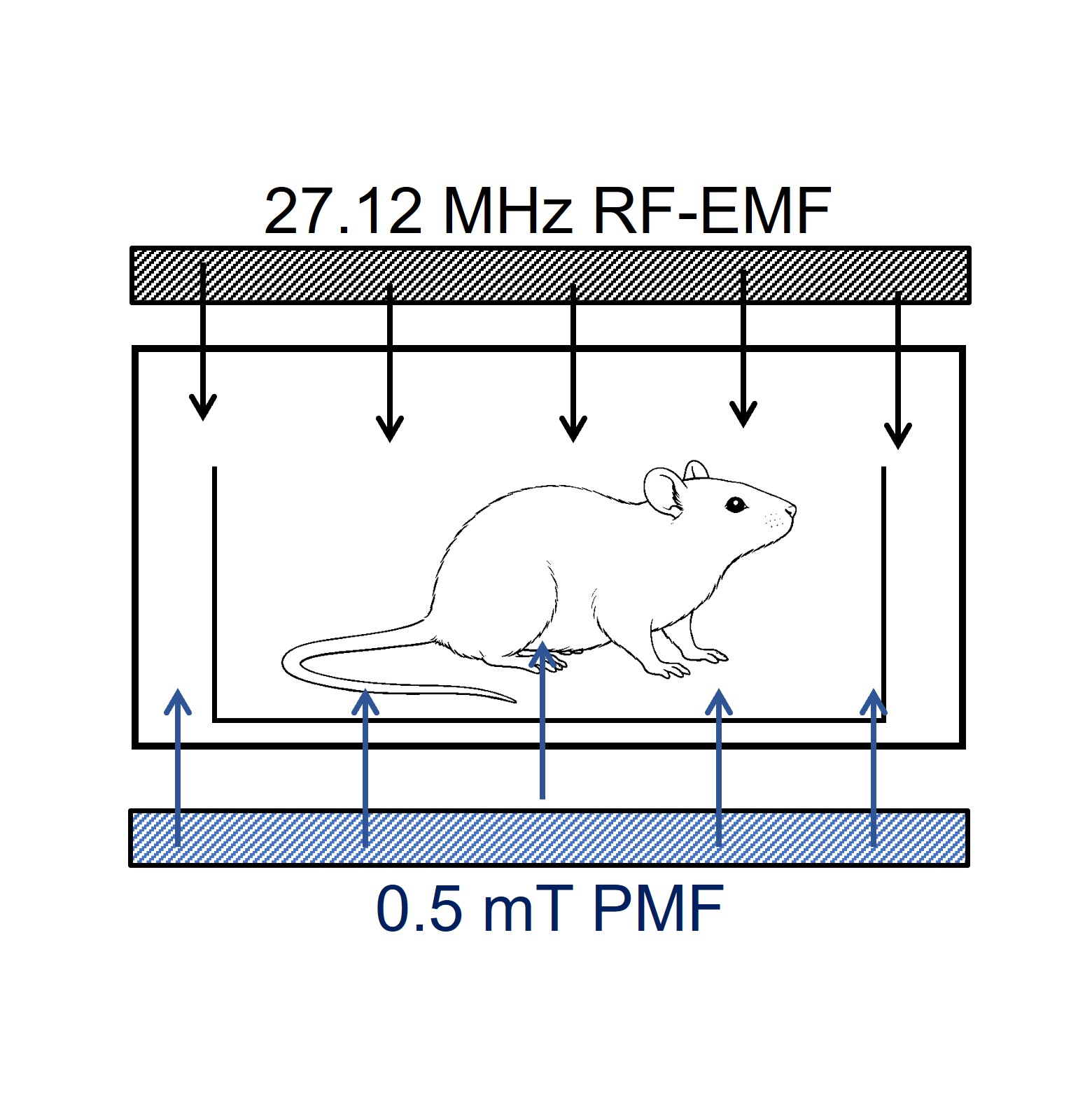

Supplement: Supplementary file 2 — Supplementary Material 2: Fig. S2: Schematic representation of the experimental setup used for electromagnetic field exposure. Animals were placed centrally within a non-conductive exposure chamber. Low-frequency pulsed magnetic field (PMF, 0.5 mT) was applied from the lower coil system, while radiofrequency electromagnetic field (RF-EMF, 27.12 MHz) exposure was delivered from the upper applicator. The configuration was designed to ensure homogeneous field exposure and to avoid physical contact or thermal effects during stimulation. [file 68_2026_3119_MOESM2_ESM.jpeg]
